# Release characteristics of NH_3_ and VOCs during short-term fermentation of municipal solid waste

**DOI:** 10.1039/d6ra04176k

**Published:** 2026-07-27

**Authors:** Jing Hu, Dehong Gong, Hui Xia, Lang Yang, ChunYu Dong, Hua Zhong, Yuhang Xie, Zheng Jian

**Affiliations:** a Electrical Engineering College, Guizhou University Guiyang 550025 China dhgong@gzu.edu.cn; b Huangmei Zhongdian Shanhe Environmental Protection Power Generation Co. China

## Abstract

To investigate the release characteristics of NH_3_ and volatile organic compounds (VOCs) during the short-term storage and fermentation of municipal solid waste, a 7-day short-term fermentation system was established using simulated municipal solid waste under 30, 40 and 50 °C conditions. NH_3_ was quantitatively monitored using an online monitoring method, while typical VOCs were qualitatively identified by GC-MS and semi-quantitatively analyzed based on normalized peak areas. The results showed that the pile temperature increased and the moisture content decreased during fermentation, indicating the continuous occurrence of microbial metabolism, moisture migration and organic matter degradation. NH_3_ concentration accumulated continuously with fermentation time, and increasing temperature significantly promoted NH_3_ release. The normalized peak-area responses of different VOC categories showed distinct variations: aromatic hydrocarbons were mainly released in the early and middle stages; halogenated hydrocarbons exhibited relatively high responses in the presence of plastic components; oxygenated VOCs increased markedly during the middle fermentation stage; alkanes were mainly characterized by continuous volatilization; and alkenes showed clear stage-dependent variations. The theoretical odor activity value (OAV) calculated from the quantitative NH_3_ concentration indicated that NH_3_ was a priority odor component requiring control during short-term fermentation. These results provide a reference for odor control, priority component screening and ventilation management in waste storage pits of municipal solid waste incineration plants.

## Abstract 2

This study investigates NH_3_ and VOC release during short-term fermentation of municipal solid waste under 30, 40 and 50 °C, using a 7-day simulated system with online NH_3_ monitoring and GC-MS analysis.

## Graphical abstract

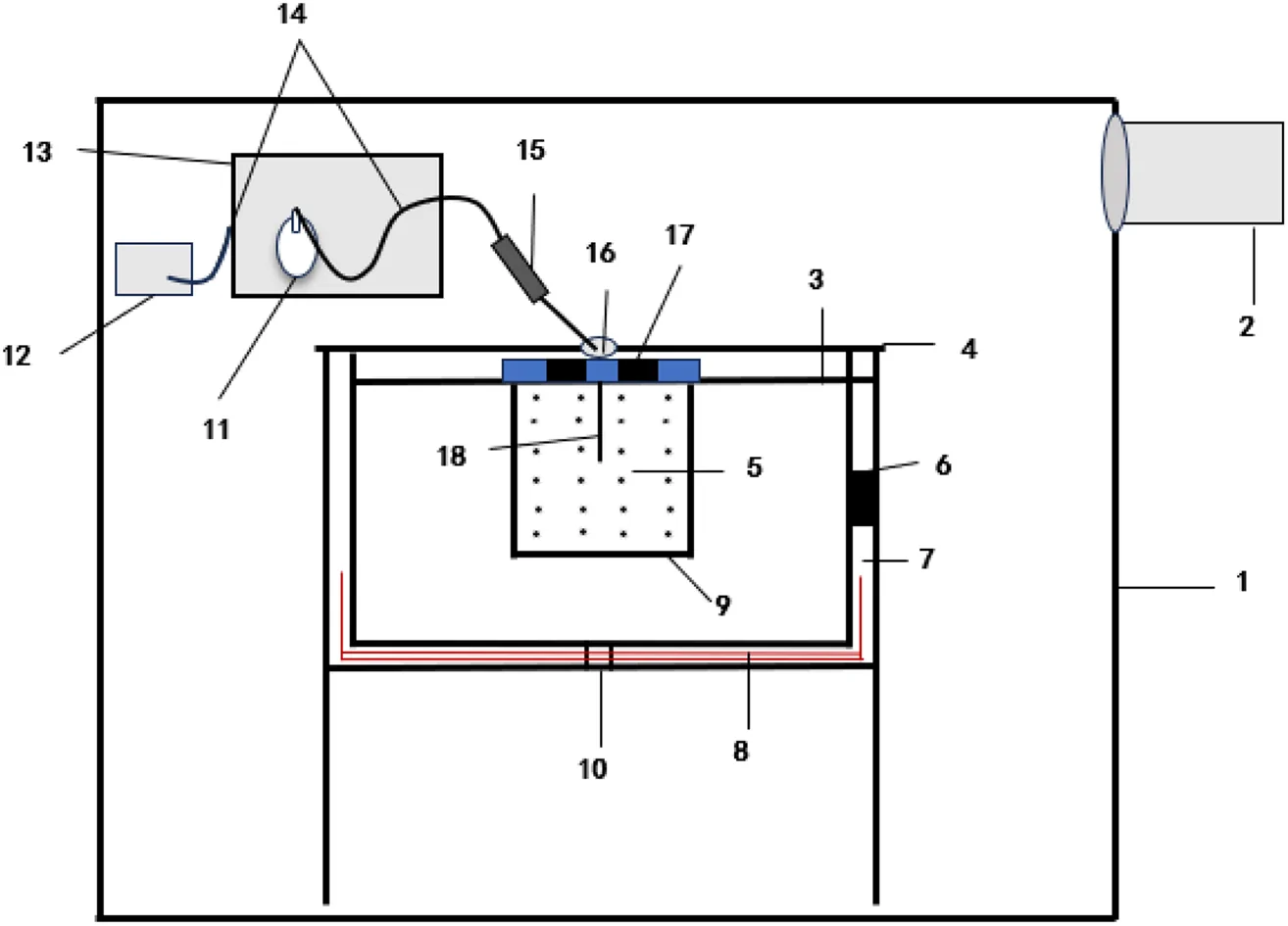

## 1 Introduction

With the continuous increase in municipal solid waste generation, waste incineration has become an important technical route for achieving waste reduction, harmless treatment and resource recovery. In practical engineering operations, municipal solid waste is usually stored in waste pits for approximately 3–5 days to improve combustion stability and calorific-value uniformity. During this process, natural fermentation and dehydration reduce the moisture content and promote the homogenization of waste components. However, complex physical migration and microbial degradation processes occur inside the waste during this stage, leading to the release of NH_3_, VOCs and other odor compounds, which may pose potential risks to the plant environment and the health of operators.^1–3^

Previous studies have shown that municipal solid waste, food waste and organic fractions can release various volatile organic compounds (VOCs) during storage, composting and biodegradation, including alcohols, ketones, esters, aromatic hydrocarbons and terpenes.^4–6^ VOCs mainly originate from the direct volatilization of volatile organic constituents and microbial metabolic processes. NH_3_ is primarily produced from the degradation and deamination of nitrogen-containing organic matter, such as proteins and amino acids, and is a common irritating inorganic odor component during the fermentation of food waste and municipal solid waste.^7,8^ In addition, due to compaction, leachate accumulation and the formation of locally oxygen-deficient zones in real waste pits, sulfur-containing odor compounds such as H_2_S, dimethyl sulfide and carbon disulfide may also be generated. Their emission intensities are closely related to oxygen supply, sulfur-containing substrates, microbial communities and fermentation stages.^9,10^

At present, studies on odor emissions from municipal solid waste have mainly focused on landfill, composting and anaerobic digestion processes.^3,5,11^ In contrast, relatively limited attention has been paid to the short-term storage stage in waste pits of municipal solid waste incineration plants, especially the simultaneous release characteristics of NH_3_ and VOCs under approximately open or weakly confined conditions. Actual waste pits are open or semi-open systems, in which gas transport, oxygen diffusion, moisture-content variation and microbial activity are highly heterogeneous. Therefore, field monitoring makes it difficult to control individual factors effectively. Constructing a simulated experimental system is helpful for clarifying the release behavior of gaseous pollutants during short-term waste storage under relatively controlled conditions.^1,12^

Based on this background, simulated municipal solid waste was used in this study to establish a short-term fermentation system under approximately open and weakly confined conditions, in order to simulate the actual 3–5 day storage process of waste in incineration plant pits. Unlike previous studies that mainly focused on long-term composting, landfill or end-of-pipe emission monitoring, this study emphasizes the coupling relationship among temperature variation, moisture-content reduction and odor emission profiles during short-term storage. On the one hand, the cumulative release of NH_3_ under different fermentation temperatures and its theoretical odor activity value were quantitatively evaluated. On the other hand, the stage-dependent release characteristics of different VOC categories were compared using normalized GC-MS peak areas. The results are expected to provide a basis for front-end ventilation control in waste pits, screening of potential priority odor-related components and subsequent quantitative verification.

## 2 Materials and methods

### 2.1 Experimental materials

According to the composition characteristics of municipal solid waste in Guiyang, four categories of waste, namely food waste, paper, wood/bamboo waste and plastics/rubber, were selected as fermentation feedstocks to prepare simulated municipal solid waste samples for odor emission experiments. The main components, mass fractions and sources of each waste category are listed in Table 1. Large and irregular food waste and paper materials were crushed using a small grinder, and then thoroughly mixed with pre-crushed wood/bamboo particles and plastic/rubber particles to obtain the final waste fermentation samples.

**Table 1:** Raw materials used for simulated municipal solid waste preparation

Category	Main components	Mass fraction	Source
Food waste	Rice, meat, vegetable leaves, eggshells and fruit peels	50%	Canteens and fruit shops at Guizhou University
Paper	Corrugated cardboard and books	20%	Express packaging cartons
Wood/bamboo waste	Sawdust and bamboo chips	10%	Wood processing factory
Plastics/rubber	PVC plastic particles	20%	Plastic processing factory

### 2.2 Experimental design

**Fig. 1:**
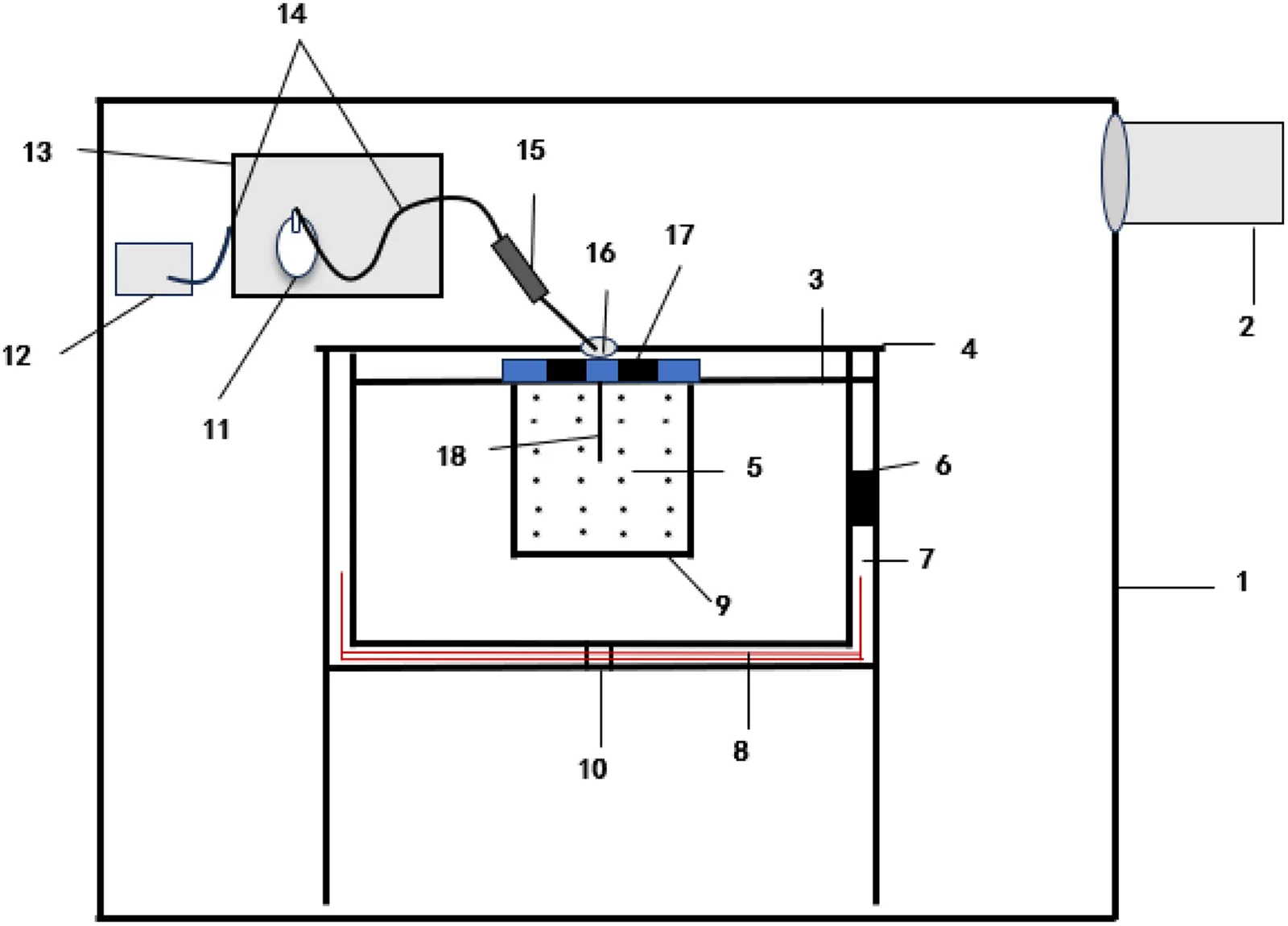
Schematic diagram of the waste fermentation experimental device. (1-Fume hood, 2-suction pump, 3-fixed bracket, 4-cover, 5-experimental garbage, 6-circulating water pump, 7-water bath layer, 8-temperature-controlled thermocouple, 9-garbage fermentation bucket, 10-outlet, 11-air bag, 12-sampling host (air pump), 13-vacuum box, 14-connecting pipe, 15-sampling gun, 16-sampling orifice, 17-temperature/humidity sensor array, 18-temperature sampling probe).

The schematic diagram of the waste fermentation system is shown in Fig. 1. Briefly, 5 kg of the prepared simulated waste was weighed using an electronic balance and loaded into a fermentation bucket, which was then placed in a temperature-controlled fermentation chamber. The fermentation temperature was set to 30, 40 or 50 °C, and the experimental period was 7 d. The main frame of the fermentation device and the effective volume of the fermentation bucket were 144 L and 50 L, respectively. A cover with reserved sampling holes was installed at the top of the device for ventilation and odor sampling. A water bath layer was used for temperature control, and the temperature-control system consisted of thermocouples, a temperature controller and a circulating water pump. The electric heater heated the water in the water bath layer, while the circulating pump promoted water circulation to ensure uniform temperature distribution within the water bath. This configuration helped maintain relatively uniform heating of the waste pile in the fermentation chamber. The temperature controller maintained the fermentation temperature within ±1 °C to ensure consistent experimental conditions and reduce the influence of external environmental fluctuations.

To investigate the release behavior of odorous substances during short-term waste fermentation, gas sampling and monitoring were carried out sequentially during the fermentation period. Sampling was conducted on days 1, 2, 3, 4, 5, 6 and 7 to capture the dynamic evolution of gas emissions. Parallel experiments were performed (*n* ≥ 2) to improve data reliability and repeatability. No turning or artificial disturbance was applied during fermentation to maintain system stability.

### 2.3 Gas sampling method

Odorous gas samples were collected according to the bag sampling method for volatile organic compounds in stationary source exhaust gas (HJ 732-2014). Before sampling, the gas sampling bag was evacuated and placed horizontally in a vacuum box. The PTFE valve of the sampling bag was connected to the sampling port of the vacuum box using a connecting tube, and the vacuum box was then sealed. Outside the vacuum box, the sampling gun and sampling pump were connected sequentially to the external interface of the vacuum box.

During sampling, the probe of the sampling gun was placed above the waste materials in the fermentation chamber. The sampling pump was started and the gas flow rate was adjusted to 1 L min^−1^. Sampling was stopped when the gas bag was filled to approximately 80% of its maximum volume of 1 L. To reduce interference caused by adsorption on the inner surface of the sampling bag, the gas bag was purged and refilled three times before formal sampling. After sampling, each gas bag was labelled and immediately transferred for instrumental analysis. The collected gas samples were analysed within a short time to avoid changes in composition caused by diffusion or chemical reactions. At least 2–3 parallel samples were collected at each sampling time point.

### 2.4 Online monitoring of NH_3_

**Fig. 2:**
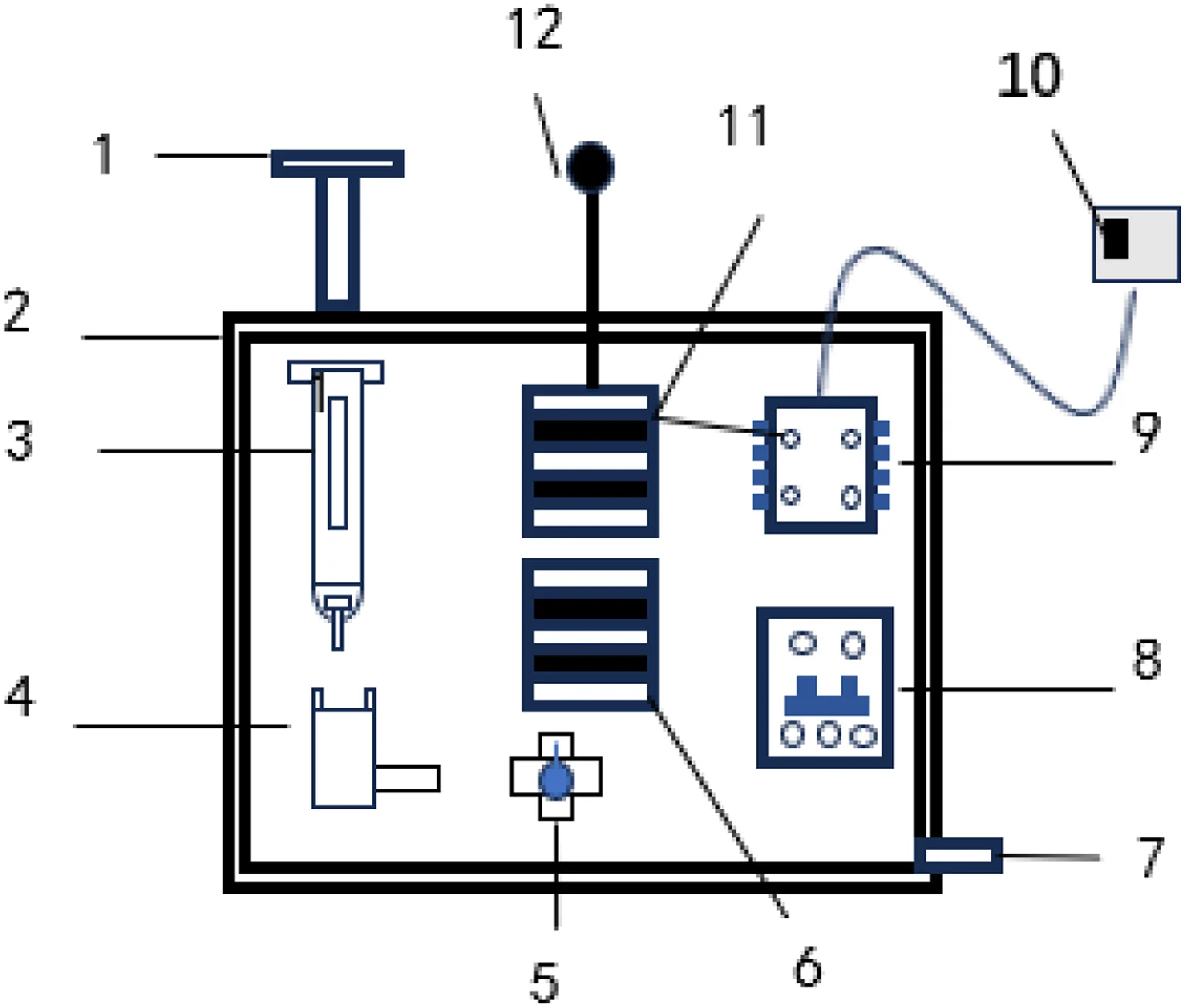
Online monitoring system for NH_3_ and gaseous pollutants. (1-Sampling probe, 2-external cabinet of the monitoring system, 3-filter, 4-air pump, 5-flowmeter, 6-gas sensor array, 7-gas outlet, 8-circuit breaker, 9-data acquisition and transmission module (data logger), 10-power supply, 11-temperature/humidity sensor, 12-temperature/humidity sampling probe).

The NH_3_ concentration was continuously measured using the online monitoring system shown in Fig. 2. The system was equipped with a sensor array consisting of electrochemical sensors and metal oxide semiconductor sensors, and temperature and humidity sensors were also integrated for multi-parameter monitoring. The system enabled real-time acquisition of NH_3_ concentration, total volatile organic compounds (TVOCs), temperature and humidity, and the recorded data were transmitted and stored through a wireless transmission module.Performance tests showed that the repeatability of NH_3_ detection was approximately 1.04%, the average relative error was approximately 2.5%, and the response time was approximately 33 s, indicating that the system met the accuracy requirements of this experiment. During fermentation, NH_3_ concentration was continuously monitored and recorded to obtain its dynamic release characteristics during waste fermentation.

### 2.5 VOC analysis method

Volatile organic compounds (VOCs) were analyzed offline using gas chromatography-mass spectrometry (GC-MS). Gas samples were introduced into the GC-MS system through gas-bag injection. It should be emphasized that the VOC results in this study were used for compound identification and semi-quantitative comparison based on normalized peak areas, rather than for absolute concentration quantification. The main analytical conditions were as follows.

The GC conditions were as follows: a DB-5MS capillary column (60 m × 0.32 mm × 1.0 µm) was used; high-purity helium (≥99.999%) was used as the carrier gas at a flow rate of 1.5 mL min^−1^; the injection mode was splitless injection; the oven temperature was initially held at 35 °C for 5 min, increased to 150 °C at 5 °C min^−1^, and then increased to 220 °C at 15 °C min^−1^ and held for 7 min; the injector temperature was 100 °C.

The MS conditions were as follows: ion source temperature, 230 °C; quadrupole temperature, 150 °C; interface temperature, 280 °C; scan range, *m*/*z* 15–400; scan rate, 5 scans per s; electron ionization energy, 70 eV; and acquisition mode, SIM/SCAN.

Compound identification was mainly performed based on retention time, mass fragment information and matching with the NIST mass spectral library. Compounds with a library matching similarity greater than 80% were considered tentatively identified. To compare the relative changes in VOC composition at different fermentation stages, normalized peak area (%) was used to represent the semi-quantitative response of each target VOC. The normalized peak area was calculated as follows:

1 *P*_*i*_ = *A*_*i*_/Σ*A*_*j*_ × 100%

where *P*_*i*_ is the normalized peak-area fraction of VOCs, *A*_*i*_ is the peak area of VOCs, and Σ*A*_*j*_ is the total peak area of all target VOCs included in the same sample. This parameter was used only to compare relative VOC emission profiles and temporal variation trends under the same analytical method, and was not used for strict comparison of absolute concentrations among different compounds.

## 3 Results and discussion

### 3.1 Evolution of temperature–humidity conditions and calorific value during waste fermentation under different temperatures

**Fig. 3:**
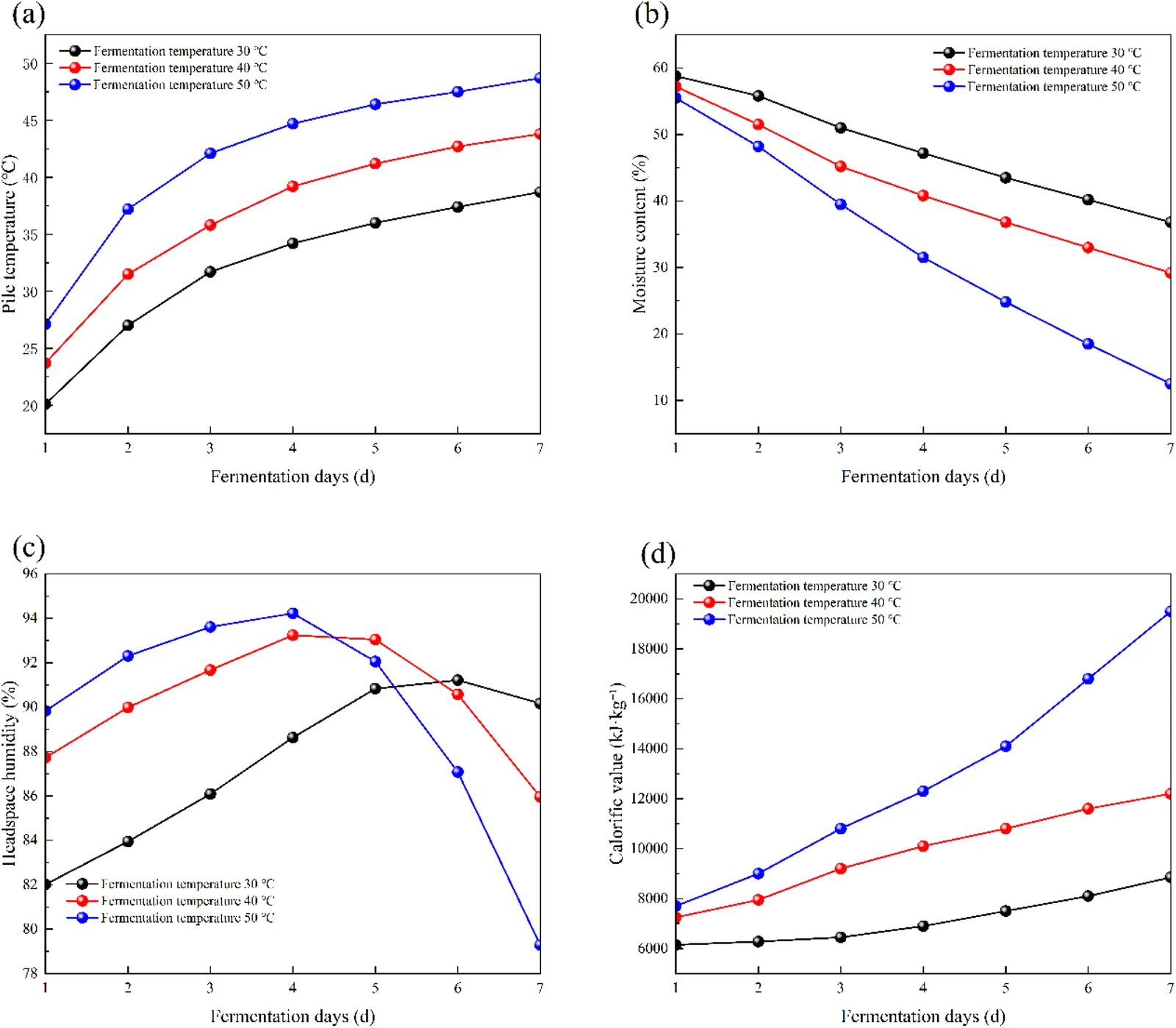
Variations in different parameters during waste fermentation under different temperatures: (a) pile temperature, (b) moisture content, (c) headspace humidity, (d) calorific value.

Fig. 3 shows the variations in pile temperature, moisture content, headspace humidity and calorific value during short-term waste fermentation under different fermentation temperatures. Overall, the waste system exhibited clear stage-dependent changes under all temperature conditions as fermentation proceeded. As shown in Fig. 3(a), the pile temperature increased continuously with fermentation time at 30, 40 and 50 °C, but the heating rate and final stable temperature differed significantly. At 30 °C, the pile temperature increased from 20.1 to 38.7 °C, showing a relatively slow warming process. At 40 °C, it increased from 23.7 to 43.8 °C. In contrast, at 50 °C, the pile temperature rose rapidly from 27.1 to 48.7 °C. In particular, all systems showed a pronounced temperature increase during the first 3 days of fermentation, indicating that microbial metabolic activity was rapidly enhanced in the early fermentation stage and that a large amount of readily degradable organic matter began to decompose and release heat. With increasing environmental temperature, both the heating rate and the final stable temperature of the waste pile increased markedly, suggesting that higher temperature promoted microbial activity and organic matter degradation inside the waste.

Fig. 3(b) shows the variation in moisture content under different fermentation temperatures. At 30 °C, the moisture content decreased from 58.8% to 36.8%; at 40 °C, it decreased from 57.2% to 29.2%; and at 50 °C, the largest decrease was observed, with the moisture content rapidly declining from 55.5% to 12.5%. These results indicate that increasing fermentation temperature enhanced water evaporation and microbial water consumption within the waste system, thereby accelerating the dehydration process. In particular, the rapid decrease in moisture content at the later stage under 50 °C demonstrates that high-temperature conditions strongly promoted moisture migration and volatilization.^13^

As shown in Fig. 3(c), the headspace humidity under different temperature conditions generally showed an initial increase followed by a decrease. At 30 °C, the headspace humidity gradually increased from 82% and remained at approximately 90% in the later stage. At 40 °C, it reached a peak during the middle fermentation stage and then began to decline. At 50 °C, the headspace humidity rapidly reached a relatively high level in the early stage but decreased markedly to below 80% in the later stage. This phenomenon indicates that enhanced evaporation of internal moisture during the early fermentation stage gradually created a high-humidity environment in the headspace. In the later stage, however, the gradual depletion of free water in the system, especially the rapid moisture loss under high-temperature conditions, led to a decrease in headspace humidity.

Fig. 3(d) presents the variation in the calorific value of waste under different fermentation temperatures. At 30 °C, the calorific value increased from 6150 to 8850 kJ kg^−1^; at 40 °C, it increased from 7250 to 12 200 kJ kg^−1^; and at 50 °C, it rose sharply from 7700 to 19 500 kJ kg^−1^. This indicates that as fermentation proceeded, the continuous decrease in moisture content resulted in the relative enrichment of combustible components per unit mass, thereby increasing the calorific value.^14^ Higher fermentation temperature further strengthened the dehydration process, and therefore the increase in calorific value was most pronounced under 50 °C.

### 3.2 NH_3_ emission characteristics

**Fig. 4:**
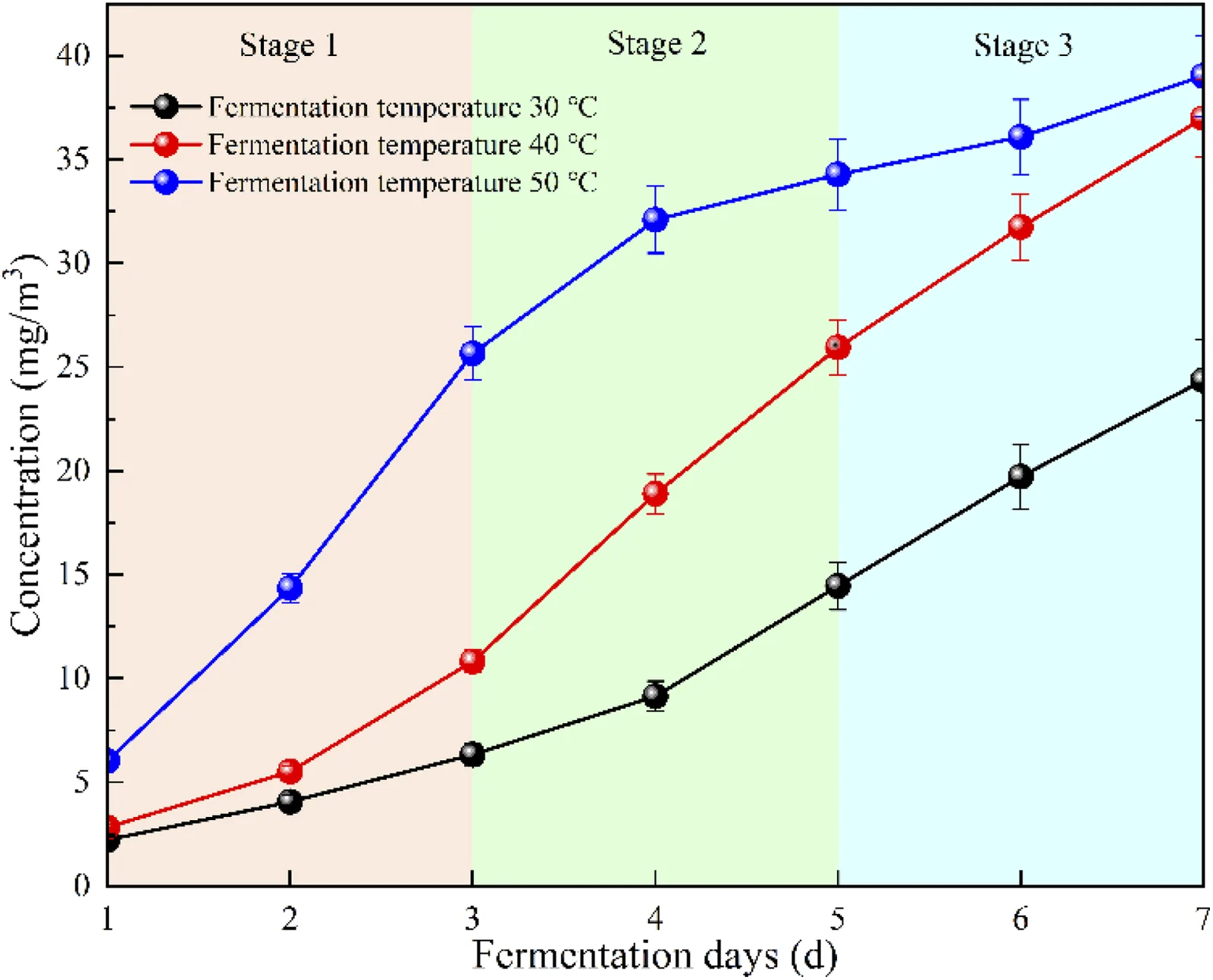
Variation in NH_3_ concentration during waste fermentation.

Fig. 4 shows the variation in NH_3_ concentration with fermentation time under different fermentation temperatures. To facilitate the analysis of the stage-dependent release characteristics of NH_3_ during short-term waste fermentation, the 7-day fermentation process was divided into three stages. Overall, NH_3_ concentration increased continuously with fermentation time under all three temperature conditions, and higher fermentation temperature led to more pronounced NH_3_ release. The NH_3_ concentration was consistently highest at 50 °C, followed by 40 °C and then 30 °C, indicating that higher temperature significantly promoted the decomposition of nitrogen-containing organic matter and the volatilization of NH_3_ from the waste.^15,16^

During stage 1 (1–3 d), the NH_3_ concentration increased rapidly. At 30 °C, NH_3_ increased from 2.4 to 6.5 mg m^−3^; at 40 °C, it increased from 3.0 to 11.0 mg m^−3^; and at 50 °C, it rose sharply from 6.1 to 25.7 mg m^−3^, showing the most pronounced increase. This suggests that, in the early fermentation stage, enhanced microbial metabolism promoted the rapid degradation of nitrogen-containing organic matter, such as proteins and amino acids, resulting in substantial NH_3_ release.^15,16^ In addition, higher temperature further intensified ammonification and NH_3_ volatilization, leading to the strongest early-stage release under 50 °C. After entering stage 2 (3–5 d), NH_3_ continued to accumulate under different temperature conditions, but the growth rates began to differ. At 30 °C, NH_3_ increased from 6.5 to 14.5 mg m^−3^; at 40 °C, it increased from 11.0 to 26.0 mg m^−3^; and at 50 °C, it increased from 25.7 to 34.4 mg m^−3^. Compared with the early stage, the increase under 50 °C became markedly slower, whereas NH_3_ still increased rapidly under 40 °C. This indicates that some readily degradable nitrogen-containing components had already been extensively released during the early stage under high-temperature conditions, while the NH_3_ release process lasted relatively longer under moderate-temperature conditions. During stage 3 (5–7 d), NH_3_ concentration further increased under all three temperature conditions, although the overall increase became smaller. The final NH_3_ concentration reached approximately 24.5 mg m^−3^ at 30 °C, 37.2 mg m^−3^ at 40 °C, and the highest value of 39.1 mg m^−3^ at 50 °C. These results indicate that organic nitrogen degradation still occurred to some extent inside the waste as fermentation proceeded. However, under high-temperature conditions, readily volatile nitrogen-containing components had gradually decreased, causing the release rate to become more moderate in the later stage.

### 3.3 Variation in the normalized peak area of aromatic hydrocarbons

**Fig. 5:**
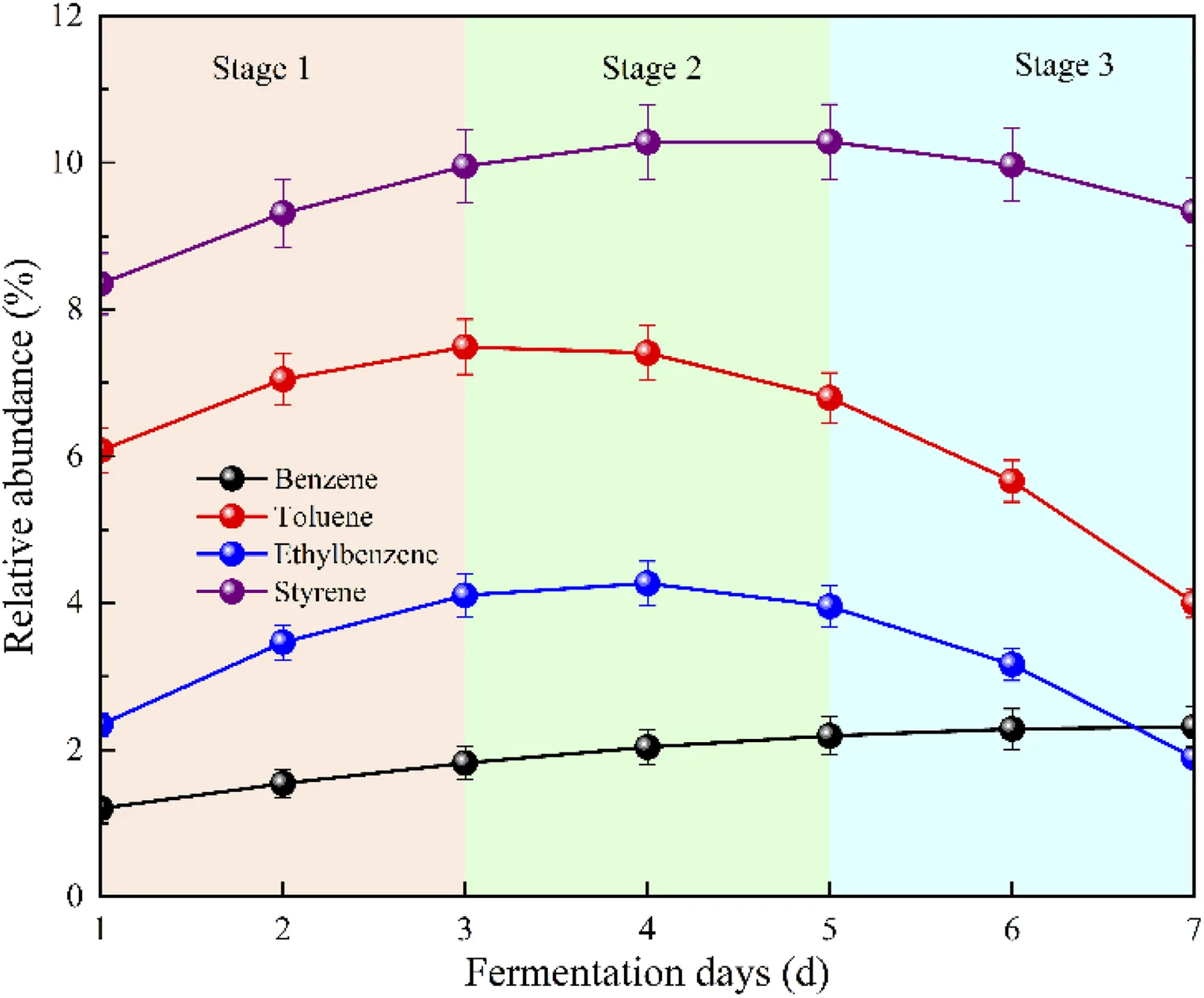
Changes in the normalized peak-area responses of aromatic hydrocarbons.

Fig. 5 shows the variation in the normalized peak area of typical aromatic hydrocarbons with fermentation time under natural storage at 30 °C. Overall, different aromatic hydrocarbon species exhibited distinct relative response patterns during fermentation. Styrene consistently showed a relatively high normalized peak area among the aromatic hydrocarbons and maintained a high relative level during the middle stage. The relative responses of toluene and ethylbenzene mainly increased during the early and middle stages and then gradually decreased in the later stage, whereas benzene showed a relatively low response with only slight variation throughout the process. These changes suggest that, during the early stage of short-term fermentation, pre-existing volatile aromatic compounds in the waste, as well as possible residual aromatic volatiles from plastics, paper and packaging materials, were first transferred into the gas phase. As fermentation proceeded, the gradual depletion of volatile precursors, together with gas-phase diffusion and microbial degradation, contributed to the decrease in the relative responses of some aromatic hydrocarbons.^17,18^

### 3.4 Variation in the normalized peak area of halogenated hydrocarbons

**Fig. 6:**
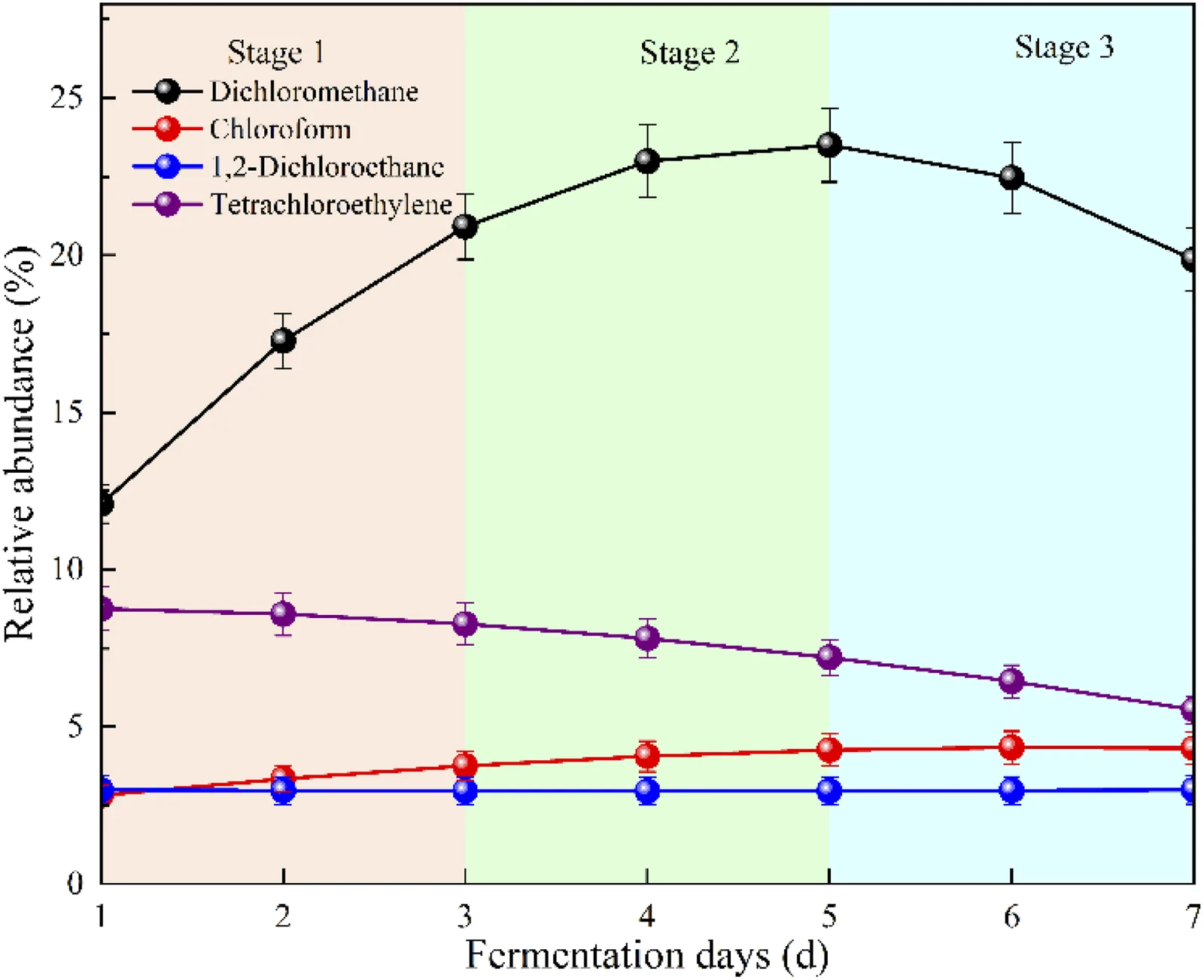
Changes in the normalized peak-area responses of halogenated hydrocarbons.

Fig. 6 shows the variation in the normalized peak area of typical halogenated hydrocarbons with fermentation time under natural storage at 30 °C. Dichloromethane exhibited a relatively high response among the halogenated hydrocarbons, reaching a high level during the early and middle stages before declining gradually. Tetrachloroethylene showed an overall decreasing trend, whereas the relative response of chloroform increased slightly with time. In contrast, 1,2-dichloroethane remained at a relatively low level throughout the fermentation process. The sources of halogenated VOCs should not be directly attributed to the thermal decomposition of the PVC main chain under the mild fermentation conditions of 30–50 °C. A more reasonable explanation is that the relative responses of halogenated hydrocarbons may be associated with residual solvents, additives, processing aids, adsorbed background volatiles from plastic particles, or background contamination in the experimental system.^18,19^ Considering the relatively high proportion of plastic/rubber components in the simulated waste used in this study, the results for halogenated VOCs should be interpreted as an indication of potential volatilization risks under plastic-containing conditions, rather than being directly extrapolated to the general emission levels of all municipal solid waste during short-term fermentation.^18–20^

### 3.5 Emission characteristics of oxygenated organic compounds

**Fig. 7:**
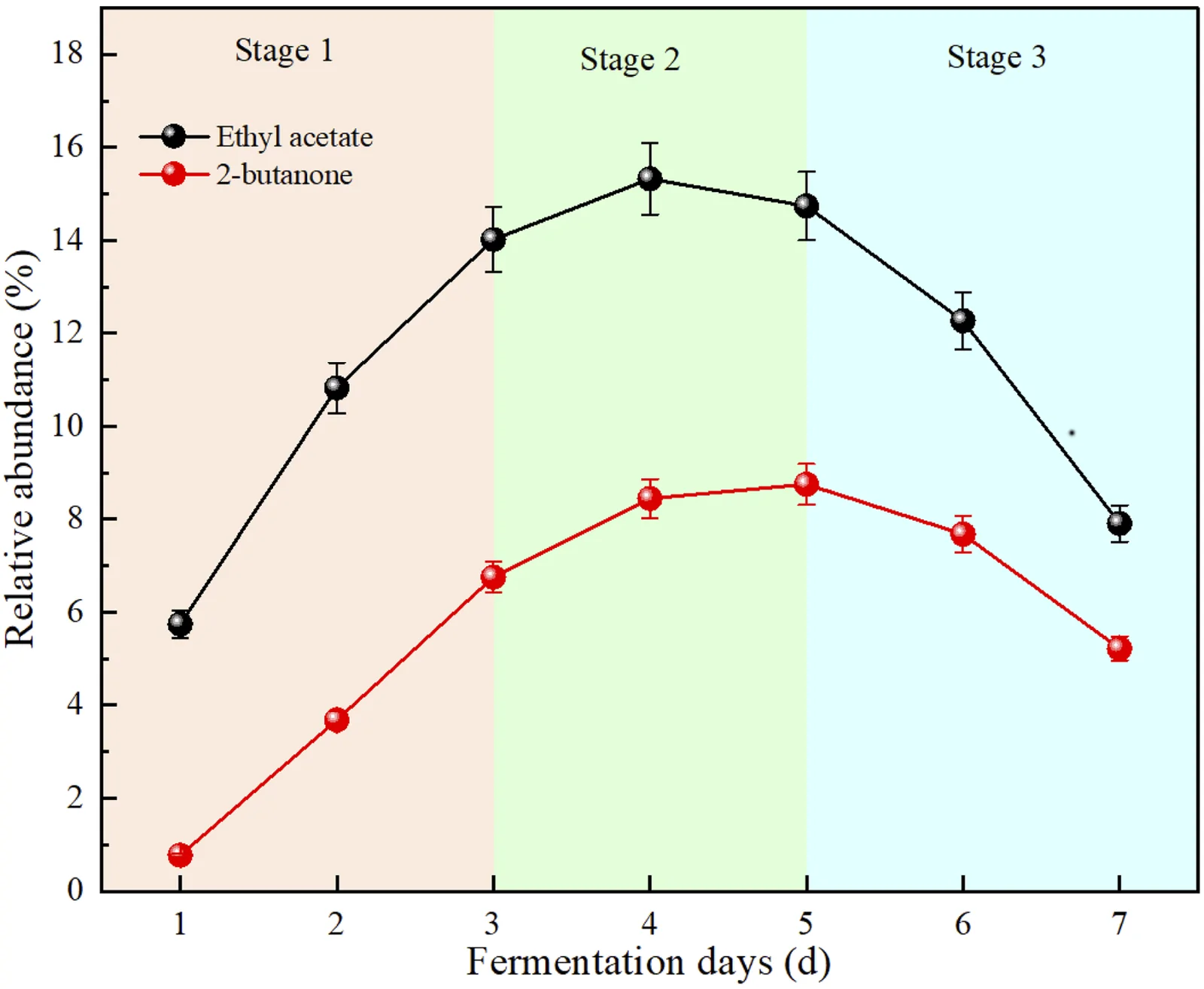
Changes in the normalized peak-area responses of oxygenated VOCs.

Fig. 7 shows the variation in the normalized peak area of typical oxygenated VOCs with fermentation time under natural storage at 30 °C. Ethyl acetate and 2-butanone both exhibited clear stage-dependent variations. The relative response of ethyl acetate increased rapidly during the early and middle stages and reached a relatively high level in the middle stage, followed by a decline. In contrast, 2-butanone showed pronounced accumulation during the middle and later stages. This phenomenon indicates that readily degradable organic matter in food waste, such as carbohydrates, starch, proteins and lipids, can be converted into intermediate products such as alcohols, organic acids and carbonyl compounds during short-term fermentation.^11,21^ These intermediates may further form ester and ketone VOCs through esterification, redox transformation or microbial metabolic processes.^11^ The relatively high response of oxygenated VOCs in the middle stage suggests that organic matter degradation and the formation of volatile metabolites were more active during this period, whereas the later-stage decrease may be associated with the depletion of volatile substrates, reduced moisture content and gas-phase diffusion loss.^18,21^

### 3.6 Variation in the normalized peak area of alkanes

**Fig. 8:**
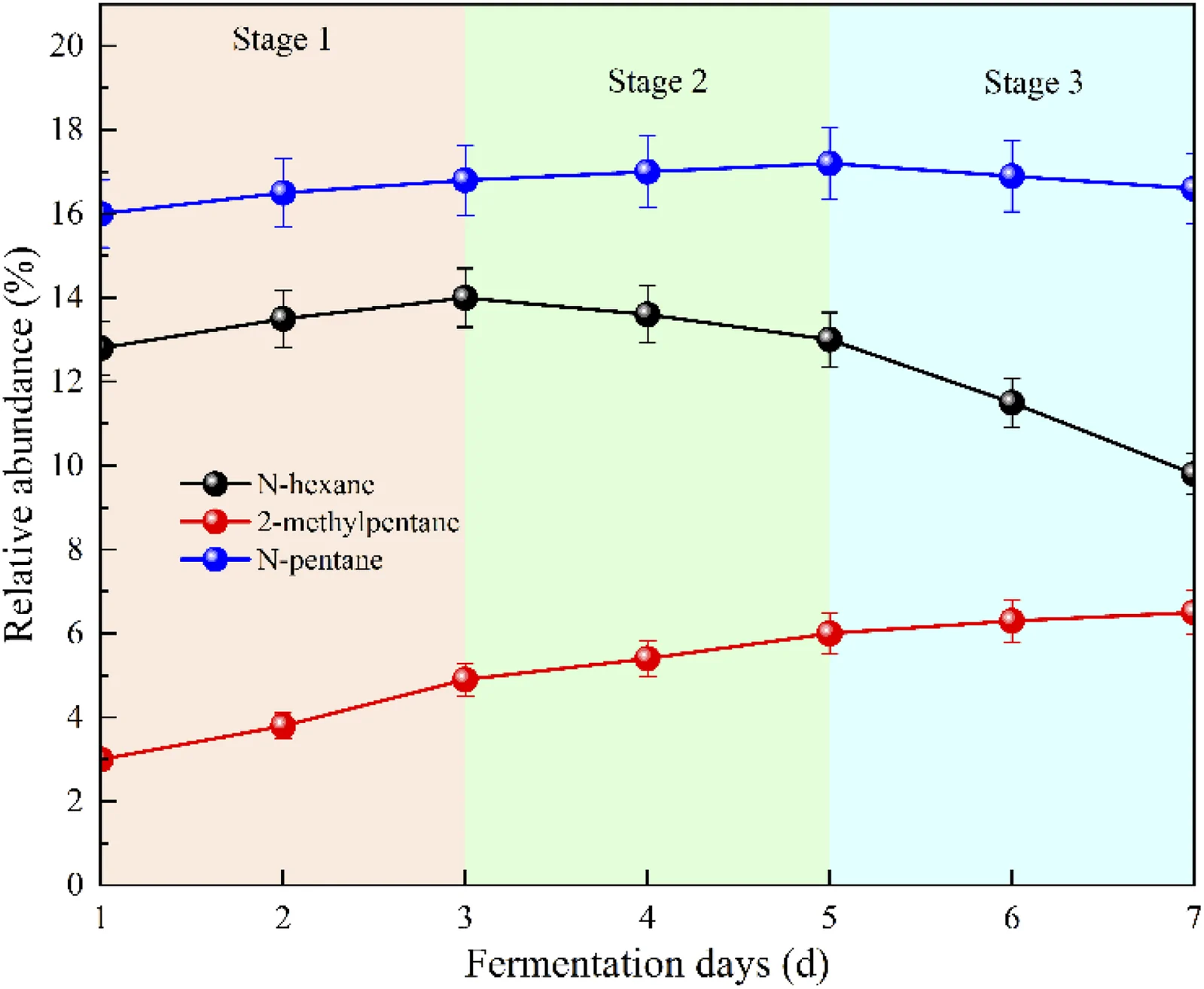
Changes in the normalized peak-area responses of alkane VOCs.

Fig. 8 shows the variation in the normalized peak area of typical alkane VOCs with fermentation time under natural storage at 30 °C. *n*-Pentane maintained a relatively high and stable peak-area response throughout the fermentation process, indicating that light alkanes exhibited a continuous volatilization-release characteristic during short-term storage. In contrast, *n*-hexane showed an increase in the early stage followed by a gradual decrease, suggesting that after the release of readily volatile components in the early stage, substrate depletion or gas-phase dissipation may have occurred. Meanwhile, 2-methylpentane displayed a pattern of gradual increase followed by stabilization. Alkane components may originate simultaneously from the physical release of low-boiling volatile compounds initially present in the waste, the migration of volatile substances associated with plastic/rubber components, as well as light hydrocarbon intermediates generated during organic matter degradation or lipid oxidation.^11,18^

### 3.7 Variation in the normalized peak area of alkenes

**Fig. 9:**
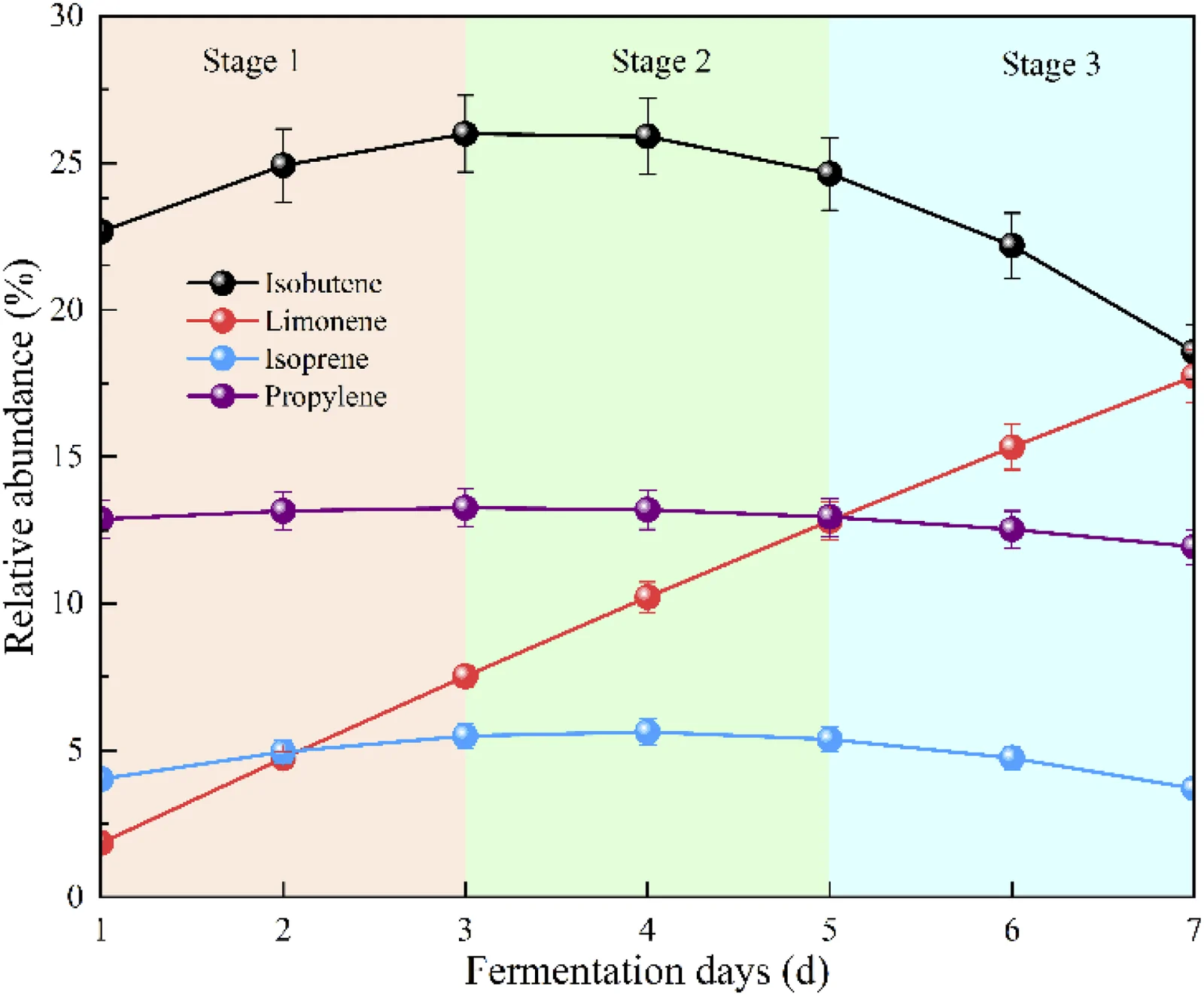
Changes in the normalized peak-area responses of alkene VOCs.

Fig. 9 shows the variation in the normalized peak area of typical alkene VOCs with fermentation time under natural storage at 30 °C. Isobutene exhibited a relatively high response during the early and middle stages, followed by a decline, indicating that light alkenes may mainly originate from the rapid release of readily volatile components in the early stage.^11^ In contrast, limonene increased continuously with fermentation time and showed a relatively high response in the later stage. Limonene is commonly associated with natural terpene components from fruit peels, plant tissues and food waste. Its later-stage accumulation may be related to the breakdown of plant tissue structures, the gradual degradation of organic matter, and enhanced gas-phase migration caused by moisture reduction. The variations in isoprene and propylene were relatively small, suggesting that their release processes were jointly influenced by substrate sources, volatility and diffusion conditions within the system. These results indicate that alkene VOCs were not released synchronously, but instead exhibited a stage-dependent differentiation characterized by the early-stage release of light alkenes and the later-stage release of terpenes.

### 3.8 Effect of fermentation temperature on the release behavior of typical VOCs

**Fig. 10:**
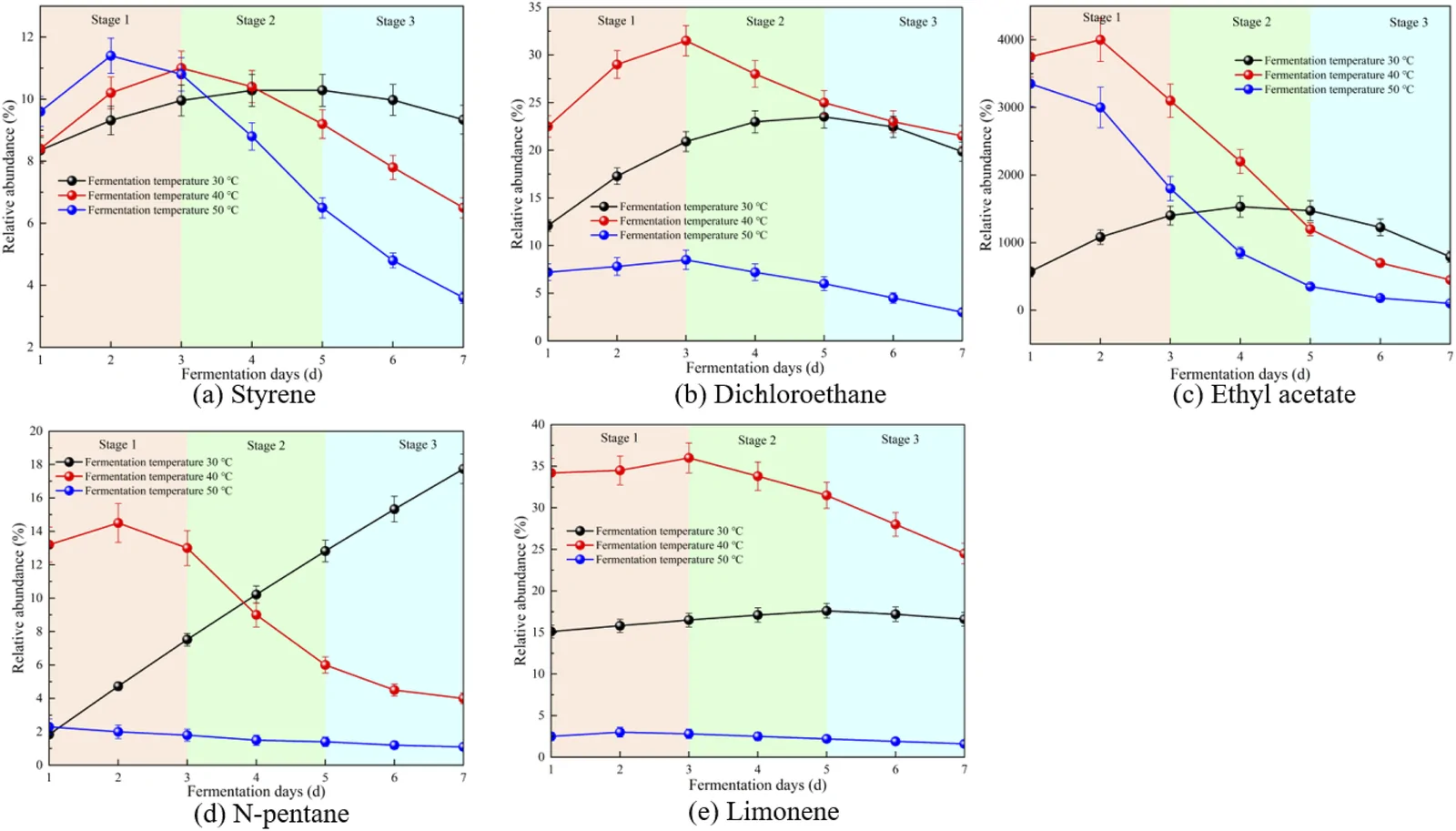
Changes in the normalized peak-area responses of typical VOCs under different fermentation temperatures (30, 40 and 50 °C): (a) styrene, (b) dichloromethane, (c) ethyl acetate, (d) limonene, (e) *n*-pentane.

To further reveal the effect of fermentation temperature on odor release behavior from waste, styrene, dichloromethane, ethyl acetate, limonene and *n*-pentane were selected as representative compounds from aromatic hydrocarbons, halogenated hydrocarbons, oxygenated VOCs, alkenes and alkanes, respectively. Their normalized peak-area variations under 30, 40 and 50 °C were compared. As shown in Fig. 10, increasing temperature did not simply lead to a continuous increase in the relative responses of all VOCs. Instead, temperature simultaneously affected substrate degradation, volatilization and migration, gas-phase diffusion, and the dissipation of readily volatile components.

For styrene, the peak appeared earlier at higher temperatures, indicating that increasing temperature promoted early-stage volatilization. However, the subsequent decrease in its relative response suggests that readily releasable components may have been rapidly dissipated in the later stage. Dichloromethane showed a relatively high response under the moderate-temperature condition, whereas its response was lower at 50 °C, suggesting a possible competition between the release of plastic-related residual volatiles and gas-phase diffusion loss.^18,19^ Ethyl acetate exhibited a high response at the early stage under 40 and 50 °C, indicating that increasing temperature promoted the rapid release of oxygenated intermediates; however, its response decreased in the later stage due to substrate depletion and moisture reduction. Limonene increased continuously at 30 °C but showed a lower relative response at higher temperatures, which may be associated with enhanced volatilization loss or accelerated degradation of terpene compounds under high-temperature conditions. *n*-Pentane showed a relatively high response during the early and middle stages at 40 °C, whereas it was difficult to maintain a high response at 50 °C, indicating that moderate-temperature conditions may be more favorable for the gas-phase accumulation of light alkanes.

### 3.9 Theoretical odor activity assessment based on quantitative NH_3_ concentration

To avoid over-interpreting the semi-quantitative VOC data, OAV values were no longer calculated for VOCs in this study. The odor activity assessment was conducted only for NH_3_ based on the quantitative concentration data obtained from online monitoring, in order to preliminarily evaluate the potential contribution of NH_3_ to odor formation during short-term waste fermentation. The OAV reflects the relationship between pollutant concentration and its odor threshold; however, it is essentially a theoretical screening indicator and cannot replace sensory validation methods such as dynamic dilution olfactometry, the triangle odor bag method, or GC-O analysis. The theoretical odor activity value of NH_3_ was calculated as follows:

2 OAV(NH_3_) = C(NH_3_)/OT(NH_3_)

where OAV(NH_3_) is the theoretical odor activity value of NH_3_, dimensionless; C(NH_3_) is the NH_3_ concentration measured during days 1–7 of fermentation; and OT(NH_3_) is the odor threshold of NH_3_, obtained from ref. 22.

**Fig. 11:**
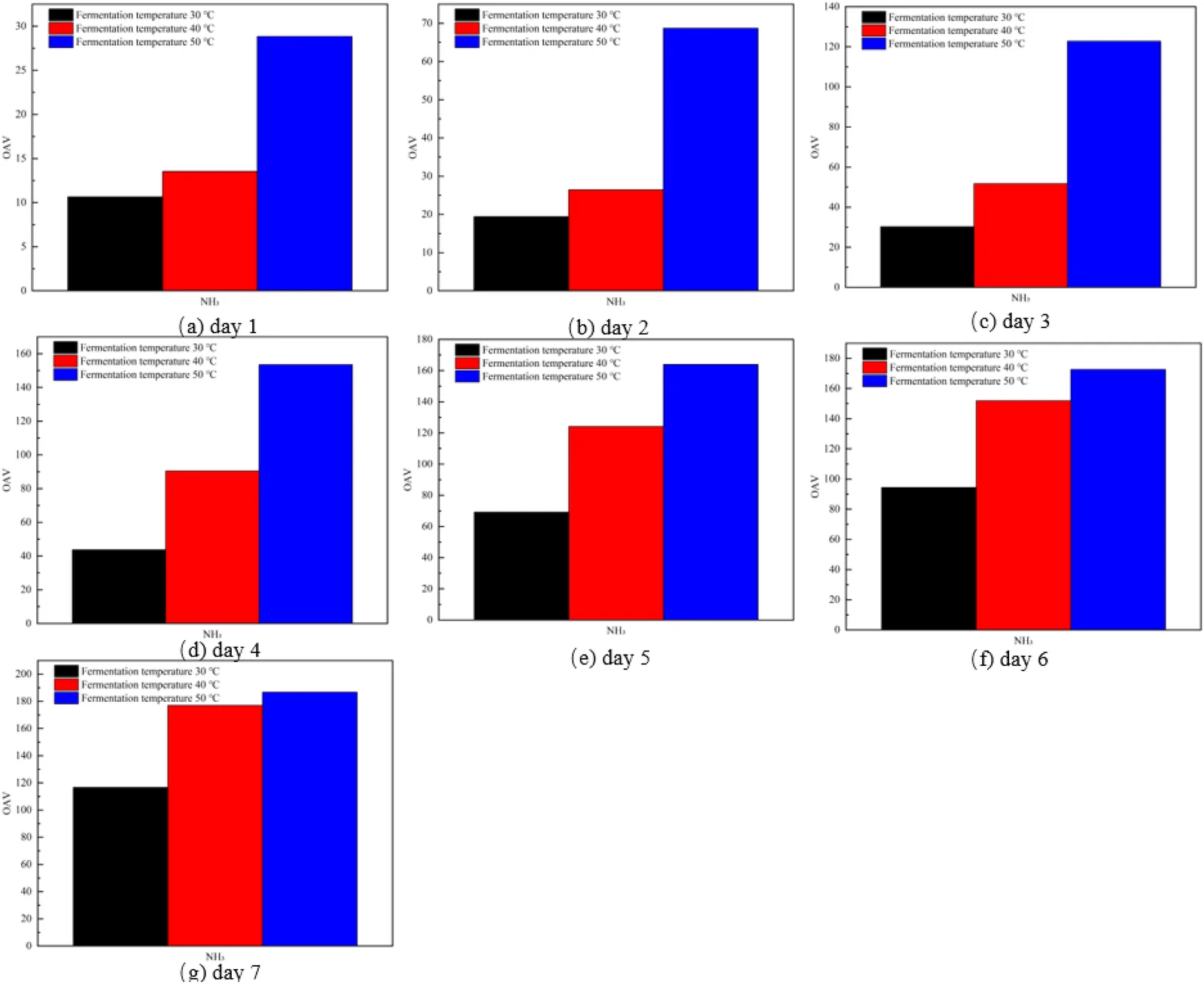
Comparison of theoretical odor activity values of NH_3_ under different fermentation temperatures (30, 40 and 50 °C) on days 1–7: (a) day 1, (b) day 2, (c) day 3, (d) day 4, (e) day 5, (f) day 6, (g) day 7.

Fig. 11 shows the variation in the theoretical odor activity value (OAV) of NH_3_ with fermentation time under different fermentation temperatures. The OAV was calculated as the ratio of the measured NH_3_ concentration to its odor threshold, and can be used to preliminarily characterize the potential contribution of NH_3_ to overall odor. It should be noted that this evaluation is a theoretical screening analysis based on concentration and odor threshold, and cannot fully replace dynamic dilution olfactometry or sensory evaluation.

Overall, the OAV of NH_3_ increased continuously with fermentation time under all three temperature conditions, and the OAV values at all stages were much higher than 1. This indicates that NH_3_ exceeded its odor threshold even in the early stage of short-term fermentation and contributed continuously to odor formation throughout the storage process. Temperature significantly enhanced the odor activity of NH_3_, with the OAV consistently following the order of 50 °C > 40 °C > 30 °C. On day 1, the OAV of NH_3_ already increased with increasing temperature. By day 3, the OAV under 50 °C had rapidly reached a high level, indicating that high temperature strongly promoted the degradation of nitrogen-containing organic matter and the volatilization of NH_3_ during the early fermentation stage. From days 4 to 7, the OAV continued to increase under all temperature conditions; however, the growth rate under 50 °C gradually slowed, while the OAV under 40 °C still showed a marked increasing trend. By day 7, the OAV values of NH_3_ under 30, 40 and 50 °C had all reached high levels, with particularly pronounced values under 40 and 50 °C.

These results indicate that NH_3_ is an inorganic odor component that requires priority attention during short-term waste storage and fermentation. Its odor contribution is affected not only by fermentation time but also by environmental temperature. Higher temperature can increase the theoretical odor activity of NH_3_ by promoting the degradation of nitrogen-containing organic matter, such as proteins and amino acids, and by enhancing the volatilization and migration of NH_3_ from the liquid/solid phase to the gas phase. However, under high-temperature conditions, readily degradable nitrogen-containing substrates were rapidly consumed in the early stage, leading to a slower increase in OAV during the later stage. In contrast, under moderate-temperature conditions, NH_3_ release lasted for a longer period, and the OAV continued to increase markedly in the later stage. Therefore, during short-term storage in waste pits of municipal solid waste incineration plants, particular attention should be paid to high-temperature conditions and the accumulation risk of NH_3_ during the middle and later storage stages. Measures such as shortening high-temperature residence time, strengthening front-end ventilation and improving negative-pressure collection should be adopted to reduce NH_3_-related odor exposure risks.

## 4 Conclusions

In this study, simulated municipal solid waste was used to establish a 7-day short-term fermentation system under 30, 40 and 50 °C. By combining online monitoring and GC-MS analysis, the quantitative release characteristics of NH_3_ and the changes in the relative VOC emission profiles during short-term waste fermentation were systematically investigated. The relationships among pile temperature, moisture content and odor release behavior were also analyzed. The results show that obvious organic matter degradation, moisture migration and volatile release occurred during short-term waste fermentation, and different odor components differed in release stage, relative response and temperature sensitivity. The main conclusions are as follows:

(1) Under natural storage at 30 °C, the pile temperature increased continuously, while the moisture content gradually decreased during waste fermentation, showing clear stage-dependent variation. Within 7 days, the pile temperature increased from 21.8 to 38.5 °C, and the moisture content decreased from 57.8% to 36.8%, indicating enhanced microbial metabolic activity accompanied by continuous water evaporation and organic matter degradation.

(2) NH_3_ accumulated continuously throughout the fermentation process and reached 24.9 mg m^−3^ on day 7. Its release followed a stage-dependent pattern characterized by rapid generation in the early stage, continuous accumulation in the middle stage and pronounced enhancement in the later stage. This indicates that ammonification was continuously strengthened during natural waste fermentation, and that the increase in pile temperature together with the decrease in moisture content promoted NH_3_ volatilization and release.

(3) Different types of VOCs showed distinct variations in normalized peak areas during short-term fermentation. Aromatic hydrocarbons were mainly released in the early and middle stages, with styrene exhibiting a relatively high response. Among the halogenated hydrocarbons, dichloromethane showed a relatively high response under plastic-containing conditions; however, its source was more likely associated with plastic-related residual volatiles, additives or background adsorbed compounds, rather than being directly attributed to PVC thermal decomposition. Oxygenated VOCs increased markedly in the middle stage, suggesting active formation of ester and ketone intermediates during fermentation metabolism. Alkanes generally showed continuous volatilization characteristics, whereas alkenes exhibited stage-dependent differentiation, characterized by the early-stage release of light alkenes and the later-stage accumulation of limonene.

(4) The theoretical OAV calculated based on quantitative NH_3_ concentration showed that NH_3_ had a clear potential odor contribution under different temperature conditions, and this contribution increased with fermentation temperature. This indicates that NH_3_ is an inorganic odor component requiring priority control in the later stage of short-term waste fermentation. Because the VOC results were based on semi-quantitative normalized peak areas, VOC–OAVs were not calculated in this study. VOCs with relatively high responses and low odor thresholds, such as styrene and limonene, were considered only as potential priority compounds for subsequent quantitative analysis and sensory verification.

## Ethical statement

All methods were carried out according to relevant guidelines and regulations.

## Author contributions

J. H.: formal analysis, investigation, writing – original draft. D. H. G.: methodology, resources, supervision, validation. H. X.: methodology, data curation. L. Y.: conceptualization of visualization. C. Y. D.: review & editing, validation. H. Z.: data analysis. Y. H. X.: review & editing. Z. J.: investigation, review & editing. All authors discussed the results and approved the article.

## Conflicts of interest

The authors declare no competing interests.

## Data Availability

Data is provided within the manuscript.
